# Crystal structure of (*E*)-3-{[2-(2,4-di­chloro­benzyl­idene)hydrazin-1-yl]carbon­yl}pyridinium chloride trihydrate

**DOI:** 10.1107/S2056989015000286

**Published:** 2015-01-10

**Authors:** J. Josephine Novina, G. Vasuki, M. Suresh, M. Syed Ali Padusha

**Affiliations:** a Department of Physics, Idhaya College for Women, Kumbakonam 1, India; b Department of Physics, Kunthavai Naachiar Govt. Arts College (W) (Autonomous), Thanjavur 7, India; c PG & Research Department of Chemistry, Jamal Mohamed College, Tiruchirappalli 20, India

**Keywords:** crystals structure, pyridinium, hydrazide group, hydrogen bonds

## Abstract

In the title hydrated salt, C_13_H_10_Cl_2_N_3_O^+^·Cl^−^·3H_2_O, the organic cation exhibits a dihedral angle of 8.26 (14)° between the mean planes of the pyridinium and benzene rings, and dihedral angles of 8.70 (15) and 15.93 (5)° between the mean planes of the hydrazide group and the benzene and pyridinium rings, respectively. In the crystal, N—H⋯O, N—H⋯Cl, C—H⋯O, C—H⋯Cl, O—H⋯O, O—H⋯N and O—H⋯Cl hydrogen bonds link the complex cations, chloride anions and solvent water mol­ecules into a three-dimensional network.

## Related literature   

For the biological activity of hydrazones, see: Kaplancikli *et al.* (2012 ▸); Babahan *et al.* (2013 ▸). For related structures, see: Novina *et al.* (2013 ▸, 2014 ▸). 
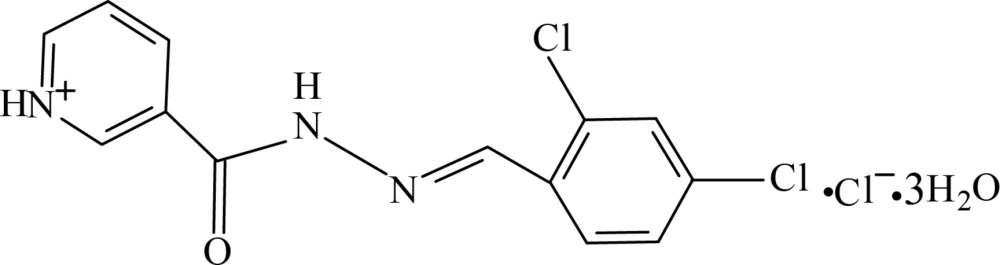


## Experimental   

### Crystal data  

C_13_H_10_Cl_2_N_3_O^+^·Cl^−^·3H_2_O
*M* *_r_* = 384.64
Triclinic, 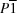

*a* = 8.4631 (4) Å
*b* = 9.5968 (5) Å
*c* = 10.8300 (6) Å
α = 76.604 (2)°
β = 89.155 (2)°
γ = 83.195 (2)°
*V* = 849.56 (8) Å^3^
*Z* = 2
Mo *K*α radiation
μ = 0.56 mm^−1^
*T* = 293 K
0.35 × 0.30 × 0.25 mm

### Data collection  

Bruker Kappa APEXII CCD diffractometer
Absorption correction: multi-scan (*SADABS*; Bruker, 2008 ▸) *T*_min_ = 0.875, *T*_max_ = 0.908
6548 measured reflections
4037 independent reflections
3007 reflections with *I* > 2σ(*I*)
*R*_int_ = 0.019

### Refinement  

*R*[*F* ^2^ > 2σ(*F* ^2^)] = 0.043
*wR*(*F* ^2^) = 0.124
*S* = 1.04
4037 reflections
234 parameters
8 restraints
H atoms treated by a mixture of independent and constrained refinement
Δρ_max_ = 0.28 e Å^−3^
Δρ_min_ = −0.37 e Å^−3^

### 

Data collection: *APEX2* (Bruker, 2008 ▸); cell refinement: *APEX2* and *SAINT* (Bruker, 2008 ▸); data reduction: *SAINT* and *XPREP* (Bruker, 2008 ▸); program(s) used to solve structure: *SIR92* (Altomare *et al.*, 1993 ▸); program(s) used to refine structure: *SHELXL97* (Sheldrick, 2008 ▸); molecular graphics: *ORTEP-3 for Windows* (Farrugia, 2012 ▸) and *Mercury* (Macrae *et al.*, 2008 ▸); software used to prepare material for publication: *PLATON* (Spek, 2009 ▸).

## Supplementary Material

Crystal structure: contains datablock(s) I, global. DOI: 10.1107/S2056989015000286/bq2398sup1.cif

Structure factors: contains datablock(s) I. DOI: 10.1107/S2056989015000286/bq2398Isup2.hkl

Supporting information file. DOI: 10.1107/S2056989015000286/bq2398Isup3.cml

**Fig. 1.**
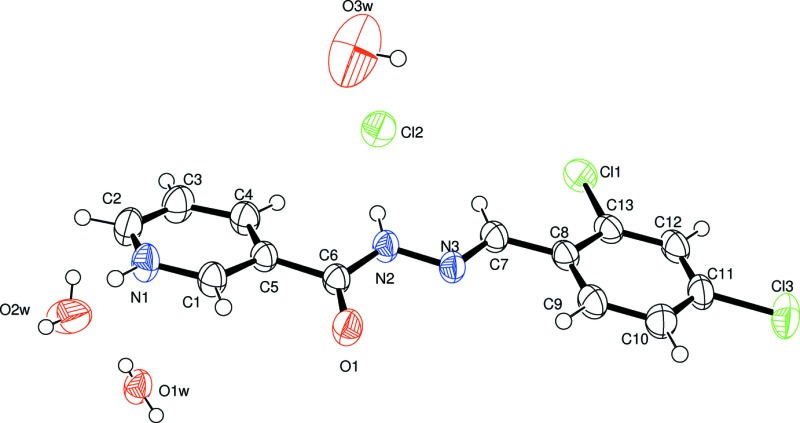
The molecular structure of the title compound, with the atom labelling. Displacement ellipsoids are drawn at the 50% probability level.

**Fig. 2.**
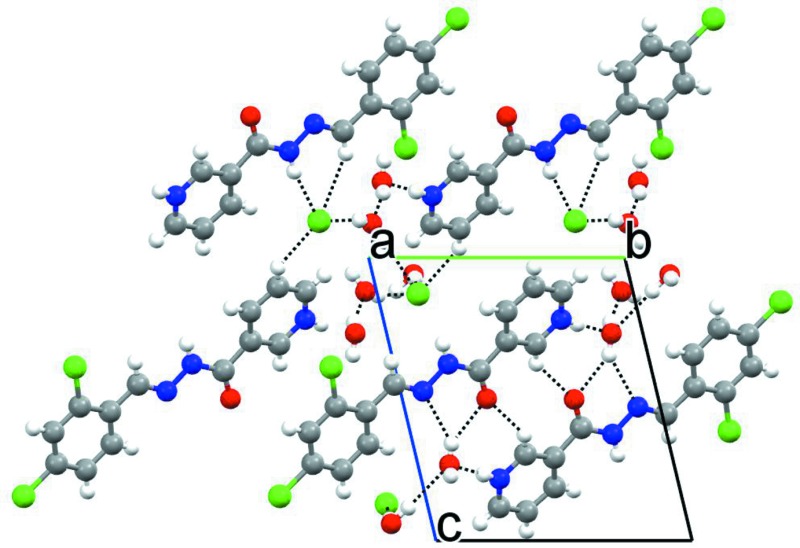
Crystal packing of the title compound viewed along the a axis. Hydrogen bonds are shown as dashed lines.

. DOI: 10.1107/S2056989015000286/bq2398fig1.tif
The mol­ecular structure of the title compound, with the atom labelling. Displacement ellipsoids are drawn at the 50% probability level.

a . DOI: 10.1107/S2056989015000286/bq2398fig2.tif
Crystal packing of the title compound viewed along the *a* axis. Hydrogen bonds are shown as dashed lines.

**Fig. 3.**
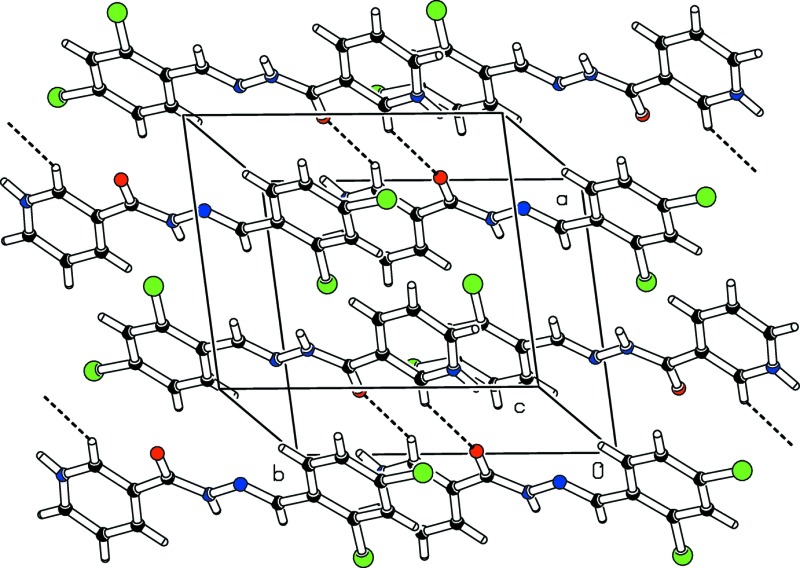
Part of the crystal packing of the title compound, showing the formation of R22(10) motif. The Cl- and the water molecules are omitted for the sake of clarity.

− . DOI: 10.1107/S2056989015000286/bq2398fig3.tif
Part of the crystal packing of the title compound, showing the formation of 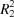
(10) motif. The Cl^−^ and the water mol­ecules are omitted for the sake of clarity.

CCDC reference: 1028701

Additional supporting information:  crystallographic information; 3D view; checkCIF report

## Figures and Tables

**Table 1:** Hydrogen-bond geometry (, )

*D*H*A*	*D*H	H*A*	*D* *A*	*D*H*A*
N1H1*N*O1*W* ^i^	0.88(2)	1.77(2)	2.646(2)	176(3)
N2H2*N*Cl2	0.86(2)	2.40(2)	3.2432(19)	168(2)
C1H1O1^ii^	0.93	2.40	3.214(3)	146
C3H3Cl2^iii^	0.93	2.79	3.608(2)	147
C7H7Cl2	0.93	2.76	3.588(2)	149
O1*W*H1*WA*O2*W*	0.77(2)	1.97(2)	2.711(3)	163(3)
O1*W*H1*WB*O1^iv^	0.81(2)	2.12(2)	2.826(2)	146(3)
O1*W*H1*WB*N3^iv^	0.81(2)	2.53(2)	3.218(2)	143(3)
O2*W*H2*WA*Cl2^iii^	0.83(2)	2.36(2)	3.190(2)	177(3)
O2*W*H2*WB*Cl2^v^	0.81(2)	2.42(2)	3.214(2)	169(3)
O3*W*H3*WA*Cl2	0.93(2)	2.28(2)	3.206(3)	174(3)
O3*W*H3*WB*O1*W* ^vi^	0.90(2)	2.25(2)	3.146(4)	177(3)

Symmetry codes: (i) 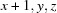; (ii) 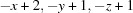; (iii) 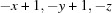; (iv) 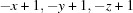; (v) 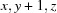; (vi) 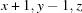.

## supplementary crystallographic information

### S1. Structural commentary

Hydro­zones have received considerable attention due to their biological importance in medicinal chemistry. Many studies have confirmed that hydrazone derivatives exhibit a wide spectrum of biological effects including anti-inflammatory activity (Kaplancikli *et al.*, 2012). Moreover, the hydrazone group plays an important role of the anti­microbial and possesses inter­esting anti­bacterial, anti­fungal and anti-tubercular activities (Babahan *et al.*, 2013). As part of our studies on hydrazone derivatives (Novina *et al.*, 2013; 2014), we report herein the crystal structure of the title compound.

The asymmetric unit of the title compound, illustrated in Fig. 1, consists of one organic cation, one Cl^-^ anion and three water molecules. The hydrazone molecule adopts an E–configuration with respect to the N3═C7 bond with the torsion angle of N2—N3—C7—C8 = -178.81 (18)°. Phenyl and pyridine rings (C8—C13 and C1/N1/C2—C5, respectively) are each planar with a dihedral angle of 8.26 (14)° between their mean-planes. The mean plane through the hydrazide unit (N3/N2/C6/O1) forms dihedral angle of 8.70 (15) and 15.93 (5)°, respectively, with the phenyl and pyridinium rings. The two chlorine atoms are in anti–periplanar positions with respect to the phenyl rings to which they are attached.

In the crystal, the organic cation, the chloride anion and the three water molecules of crystallization are linked through an intricate hydrogen-bonding network consisting of N—H···O, N—H···Cl, C—H···O, C—H···Cl, O—H···O, O—H···N and O—H···Cl inter­actions that consolidate a three–dimensional network (Table 1). One of the H atoms of the water molecule (O1W) forms bifurcated hydrogen bonds to the azomethine nitro­gen and the carbonyl oxygen atoms of one neighbouring molecule and the same water molecule acts as a hydrogen bond acceptor towards another hydrazone molecule through N–H···O hydrogen bonds (Fig. 2). Further molecules are linked via a pair of C—H···O hydrogen bonds forming inversion dimers with an *R*^2^_2_(10) ring motif (Fig. 3). The crystal structure is further stabilized by N—H···Cl, C—H···Cl and O—H···Cl hydrogen bonds.

### S2. Synthesis and crystallization

2,4-di­chloro­benzaldehyde (0.175 g, 0.001 mol) was added to an aqueous solution of nicotinicacid hydrazide (0.34 g, 0.001 mol), followed by 2 drops of concentrated HCl is added. After the addition was complete, the reaction mixture was stirred well at room temperature for 1 h. The colourless solid that formed was filtered, dried and washed with petroleum ether (40–60%). The crude solid obtained was dried and recrystallized from absolute alcohol. The recrystallized product was dried over vacuum. [m.pt: 411–413 K; yield:92%].

### S3. Refinement

The H atoms of the solvent water were located in a difference map and refined freely. All Other H atoms were placed in geometrically idealized positions and constrained to ride on their parent atoms with C—H = 0.93 Å, N—H = 0.86–0.88 Å and with *U*_iso_(H) = 1.2*U*_eq_(CH).

### Crystal data

**Table:**

C_13_H_10_Cl_2_N_3_O^+^·Cl^−^·3H_2_O	*Z* = 2
*M**_r_* = 384.64	*F*(000) = 396
Triclinic, *P*1	*D*_x_ = 1.504 Mg m^−^^3^
*a* = 8.4631 (4) Å	Mo *K*α radiation, λ = 0.71073 Å
*b* = 9.5968 (5) Å	Cell parameters from 6548 reflections
*c* = 10.8300 (6) Å	θ = 1.0–28.2°
α = 76.604 (2)°	µ = 0.56 mm^−^^1^
β = 89.155 (2)°	*T* = 293 K
γ = 83.195 (2)°	Block, colorless
*V* = 849.56 (8) Å^3^	0.35 × 0.30 × 0.25 mm

### Data collection

**Table:**

Bruker Kappa APEXII CCD diffractometer	4037 independent reflections
Radiation source: fine-focus sealed tube	3007 reflections with *I* > 2σ(*I*)
Graphite monochromator	*R*_int_ = 0.019
ω and φ scan	θ_max_ = 28.2°, θ_min_ = 3.1°
Absorption correction: multi-scan (*SADABS*; Bruker, 2008)	*h* = −11→11
*T*_min_ = 0.875, *T*_max_ = 0.908	*k* = −9→12
6548 measured reflections	*l* = −8→14

### Refinement

**Table:**

Refinement on *F*^2^	Primary atom site location: structure-invariant direct methods
Least-squares matrix: full	Secondary atom site location: difference Fourier map
*R*[*F*^2^ > 2σ(*F*^2^)] = 0.043	Hydrogen site location: inferred from neighbouring sites
*wR*(*F*^2^) = 0.124	H atoms treated by a mixture of independent and constrained refinement
*S* = 1.04	*w* = 1/[σ^2^(*F*_o_^2^) + (0.0621*P*)^2^ + 0.184*P*] where *P* = (*F*_o_^2^ + 2*F*_c_^2^)/3
4037 reflections	(Δ/σ)_max_ < 0.001
234 parameters	Δρ_max_ = 0.28 e Å^−^^3^
8 restraints	Δρ_min_ = −0.37 e Å^−^^3^

### Special details

**Table:**

Geometry. All e.s.d.'s (except the e.s.d. in the dihedral angle between two l.s. planes) are estimated using the full covariance matrix. The cell e.s.d.'s are taken into account individually in the estimation of e.s.d.'s in distances, angles and torsion angles; correlations between e.s.d.'s in cell parameters are only used when they are defined by crystal symmetry. An approximate (isotropic) treatment of cell e.s.d.'s is used for estimating e.s.d.'s involving l.s. planes.
Refinement. Refinement of *F*^2^ against ALL reflections. The weighted *R*-factor *wR* and goodness of fit *S* are based on *F*^2^, conventional *R*-factors *R* are based on *F*, with *F* set to zero for negative *F*^2^. The threshold expression of *F*^2^ > σ(*F*^2^) is used only for calculating *R*-factors(gt) *etc*. and is not relevant to the choice of reflections for refinement. *R*-factors based on *F*^2^ are statistically about twice as large as those based on *F*, and *R*- factors based on ALL data will be even larger.

### Fractional atomic coordinates and isotropic or equivalent isotropic displacement parameters (Å^2^)

**Table:**

	*x*	*y*	*z*	*U*_iso_*/*U*_eq_	
Cl1	0.52954 (7)	−0.24772 (6)	0.38718 (6)	0.04957 (17)	
Cl3	0.72649 (9)	−0.56838 (6)	0.84324 (7)	0.0656 (2)	
O1	0.8879 (2)	0.32962 (16)	0.49886 (15)	0.0485 (4)	
N1	0.8808 (2)	0.69456 (19)	0.21816 (19)	0.0445 (4)	
N2	0.7666 (2)	0.21144 (17)	0.37617 (17)	0.0372 (4)	
N3	0.7793 (2)	0.08783 (17)	0.47208 (16)	0.0367 (4)	
C1	0.8932 (2)	0.5703 (2)	0.3054 (2)	0.0386 (5)	
H1	0.9622	0.5563	0.3744	0.046*	
C2	0.7810 (3)	0.7223 (2)	0.1195 (2)	0.0491 (6)	
H2	0.7734	0.8112	0.0617	0.059*	
C3	0.6892 (3)	0.6188 (3)	0.1031 (2)	0.0501 (6)	
H3	0.6191	0.6371	0.0344	0.060*	
C4	0.7022 (3)	0.4873 (2)	0.1899 (2)	0.0421 (5)	
H4	0.6426	0.4157	0.1785	0.050*	
C5	0.8039 (2)	0.4620 (2)	0.29380 (19)	0.0340 (4)	
C6	0.8238 (2)	0.3282 (2)	0.39897 (19)	0.0343 (4)	
C7	0.7166 (3)	−0.0174 (2)	0.4476 (2)	0.0392 (5)	
H7	0.6685	−0.0079	0.3690	0.047*	
C8	0.7204 (2)	−0.1530 (2)	0.5436 (2)	0.0364 (4)	
C9	0.8056 (3)	−0.1751 (2)	0.6573 (2)	0.0423 (5)	
H9	0.8619	−0.1024	0.6711	0.051*	
C10	0.8088 (3)	−0.3016 (2)	0.7499 (2)	0.0450 (5)	
H10	0.8654	−0.3141	0.8254	0.054*	
C11	0.7259 (3)	−0.4091 (2)	0.7274 (2)	0.0436 (5)	
C12	0.6423 (3)	−0.3936 (2)	0.6166 (2)	0.0439 (5)	
H12	0.5886	−0.4679	0.6028	0.053*	
C13	0.6391 (2)	−0.2655 (2)	0.5254 (2)	0.0373 (4)	
Cl2	0.59217 (8)	0.15917 (7)	0.12886 (6)	0.05501 (18)	
O1W	0.0683 (2)	0.87717 (19)	0.26788 (17)	0.0547 (4)	
O2W	0.3189 (3)	0.9558 (3)	0.1201 (2)	0.0859 (7)	
O3W	0.9617 (3)	0.1595 (4)	0.0595 (4)	0.1333 (13)	
H1N	0.947 (3)	0.753 (3)	0.234 (3)	0.066 (8)*	
H2N	0.725 (3)	0.210 (3)	0.3047 (18)	0.051 (7)*	
H1WA	0.145 (3)	0.882 (4)	0.230 (3)	0.076*	
H1WB	0.088 (4)	0.849 (3)	0.3430 (18)	0.076*	
H2WA	0.344 (4)	0.923 (3)	0.057 (2)	0.076*	
H2WB	0.389 (3)	1.001 (3)	0.133 (3)	0.076*	
H3WA	0.853 (2)	0.161 (3)	0.074 (3)	0.076*	
H3WB	0.991 (4)	0.077 (2)	0.117 (3)	0.076*	

### Atomic displacement parameters (Å^2^)

**Table:**

	*U*^11^	*U*^22^	*U*^33^	*U*^12^	*U*^13^	*U*^23^
Cl1	0.0565 (3)	0.0471 (3)	0.0514 (3)	−0.0168 (2)	−0.0045 (3)	−0.0186 (2)
Cl3	0.0796 (4)	0.0369 (3)	0.0704 (4)	−0.0098 (3)	0.0025 (3)	0.0091 (3)
O1	0.0700 (10)	0.0389 (8)	0.0384 (8)	−0.0215 (7)	−0.0140 (7)	−0.0039 (6)
N1	0.0479 (10)	0.0351 (9)	0.0495 (11)	−0.0170 (8)	−0.0014 (9)	−0.0017 (8)
N2	0.0479 (10)	0.0294 (8)	0.0344 (9)	−0.0117 (7)	−0.0069 (8)	−0.0040 (7)
N3	0.0443 (9)	0.0282 (8)	0.0368 (9)	−0.0086 (7)	−0.0015 (7)	−0.0039 (7)
C1	0.0400 (10)	0.0347 (10)	0.0411 (11)	−0.0117 (8)	−0.0036 (9)	−0.0047 (9)
C2	0.0593 (14)	0.0387 (11)	0.0442 (12)	−0.0108 (10)	−0.0020 (11)	0.0034 (10)
C3	0.0572 (13)	0.0476 (12)	0.0412 (12)	−0.0078 (10)	−0.0140 (10)	0.0001 (10)
C4	0.0476 (12)	0.0382 (10)	0.0414 (11)	−0.0117 (9)	−0.0078 (9)	−0.0073 (9)
C5	0.0379 (10)	0.0297 (9)	0.0352 (10)	−0.0089 (8)	−0.0017 (8)	−0.0065 (8)
C6	0.0383 (10)	0.0304 (9)	0.0347 (10)	−0.0094 (8)	−0.0026 (8)	−0.0059 (8)
C7	0.0491 (11)	0.0307 (10)	0.0395 (11)	−0.0106 (8)	−0.0033 (9)	−0.0085 (8)
C8	0.0415 (10)	0.0280 (9)	0.0414 (11)	−0.0071 (8)	0.0027 (9)	−0.0098 (8)
C9	0.0498 (12)	0.0315 (10)	0.0479 (12)	−0.0115 (9)	−0.0007 (10)	−0.0103 (9)
C10	0.0505 (12)	0.0389 (11)	0.0446 (12)	−0.0062 (9)	−0.0015 (10)	−0.0071 (9)
C11	0.0498 (12)	0.0268 (9)	0.0509 (13)	−0.0043 (9)	0.0075 (10)	−0.0027 (9)
C12	0.0501 (12)	0.0295 (10)	0.0557 (14)	−0.0133 (9)	0.0073 (10)	−0.0133 (9)
C13	0.0407 (10)	0.0320 (10)	0.0427 (11)	−0.0084 (8)	0.0028 (9)	−0.0141 (9)
Cl2	0.0667 (4)	0.0567 (4)	0.0458 (3)	−0.0209 (3)	−0.0107 (3)	−0.0128 (3)
O1W	0.0705 (12)	0.0484 (9)	0.0457 (10)	−0.0255 (9)	−0.0100 (9)	−0.0022 (8)
O2W	0.1004 (17)	0.1131 (19)	0.0687 (14)	−0.0676 (15)	0.0228 (12)	−0.0443 (13)
O3W	0.0783 (17)	0.141 (3)	0.150 (3)	−0.0212 (18)	−0.0129 (18)	0.034 (2)

### Geometric parameters (Å, º)

**Table:**

Cl1—C13	1.736 (2)	C5—C6	1.501 (3)
Cl3—C11	1.738 (2)	C7—C8	1.463 (3)
O1—C6	1.221 (2)	C7—H7	0.9300
N1—C2	1.330 (3)	C8—C9	1.397 (3)
N1—C1	1.333 (3)	C8—C13	1.398 (3)
N1—H1N	0.882 (17)	C9—C10	1.382 (3)
N2—C6	1.346 (2)	C9—H9	0.9300
N2—N3	1.378 (2)	C10—C11	1.382 (3)
N2—H2N	0.856 (16)	C10—H10	0.9300
N3—C7	1.275 (2)	C11—C12	1.371 (3)
C1—C5	1.385 (3)	C12—C13	1.384 (3)
C1—H1	0.9300	C12—H12	0.9300
C2—C3	1.374 (3)	O1W—H1WA	0.766 (17)
C2—H2	0.9300	O1W—H1WB	0.809 (18)
C3—C4	1.381 (3)	O2W—H2WA	0.829 (17)
C3—H3	0.9300	O2W—H2WB	0.805 (17)
C4—C5	1.385 (3)	O3W—H3WA	0.930 (17)
C4—H4	0.9300	O3W—H3WB	0.897 (17)
			
C2—N1—C1	122.53 (18)	N3—C7—C8	119.86 (19)
C2—N1—H1N	125.4 (19)	N3—C7—H7	120.1
C1—N1—H1N	112.1 (19)	C8—C7—H7	120.1
C6—N2—N3	117.78 (17)	C9—C8—C13	117.38 (18)
C6—N2—H2N	123.2 (16)	C9—C8—C7	121.09 (18)
N3—N2—H2N	119.0 (16)	C13—C8—C7	121.53 (19)
C7—N3—N2	115.19 (18)	C10—C9—C8	121.99 (19)
N1—C1—C5	120.32 (19)	C10—C9—H9	119.0
N1—C1—H1	119.8	C8—C9—H9	119.0
C5—C1—H1	119.8	C11—C10—C9	118.3 (2)
N1—C2—C3	119.7 (2)	C11—C10—H10	120.9
N1—C2—H2	120.1	C9—C10—H10	120.9
C3—C2—H2	120.1	C12—C11—C10	121.94 (19)
C2—C3—C4	119.3 (2)	C12—C11—Cl3	118.97 (16)
C2—C3—H3	120.3	C10—C11—Cl3	119.09 (19)
C4—C3—H3	120.3	C11—C12—C13	118.96 (19)
C3—C4—C5	120.05 (19)	C11—C12—H12	120.5
C3—C4—H4	120.0	C13—C12—H12	120.5
C5—C4—H4	120.0	C12—C13—C8	121.4 (2)
C1—C5—C4	118.05 (18)	C12—C13—Cl1	117.93 (15)
C1—C5—C6	115.94 (17)	C8—C13—Cl1	120.64 (16)
C4—C5—C6	125.98 (17)	H1WA—O1W—H1WB	110 (3)
O1—C6—N2	123.58 (18)	H2WA—O2W—H2WB	108 (3)
O1—C6—C5	120.06 (17)	H3WA—O3W—H3WB	96 (3)
N2—C6—C5	116.35 (17)		
			
C6—N2—N3—C7	176.82 (19)	N3—C7—C8—C9	−6.6 (3)
C2—N1—C1—C5	1.9 (3)	N3—C7—C8—C13	173.4 (2)
C1—N1—C2—C3	−1.8 (4)	C13—C8—C9—C10	−0.9 (3)
N1—C2—C3—C4	0.0 (4)	C7—C8—C9—C10	179.1 (2)
C2—C3—C4—C5	1.6 (4)	C8—C9—C10—C11	0.6 (3)
N1—C1—C5—C4	−0.2 (3)	C9—C10—C11—C12	0.3 (3)
N1—C1—C5—C6	−178.47 (19)	C9—C10—C11—Cl3	−179.30 (18)
C3—C4—C5—C1	−1.6 (3)	C10—C11—C12—C13	−1.0 (3)
C3—C4—C5—C6	176.5 (2)	Cl3—C11—C12—C13	178.67 (16)
N3—N2—C6—O1	1.0 (3)	C11—C12—C13—C8	0.7 (3)
N3—N2—C6—C5	−178.36 (17)	C11—C12—C13—Cl1	−178.90 (17)
C1—C5—C6—O1	14.8 (3)	C9—C8—C13—C12	0.3 (3)
C4—C5—C6—O1	−163.3 (2)	C7—C8—C13—C12	−179.7 (2)
C1—C5—C6—N2	−165.78 (19)	C9—C8—C13—Cl1	179.82 (16)
C4—C5—C6—N2	16.1 (3)	C7—C8—C13—Cl1	−0.2 (3)
N2—N3—C7—C8	−178.81 (18)		

### Hydrogen-bond geometry (Å, º)

**Table:**

*D*—H···*A*	*D*—H	H···*A*	*D*···*A*	*D*—H···*A*
N1—H1*N*···O1*W*^i^	0.88 (2)	1.77 (2)	2.646 (2)	176 (3)
N2—H2*N*···Cl2	0.86 (2)	2.40 (2)	3.2432 (19)	168 (2)
C1—H1···O1^ii^	0.93	2.40	3.214 (3)	146
C3—H3···Cl2^iii^	0.93	2.79	3.608 (2)	147
C7—H7···Cl2	0.93	2.76	3.588 (2)	149
O1*W*—H1*WA*···O2*W*	0.77 (2)	1.97 (2)	2.711 (3)	163 (3)
O1*W*—H1*WB*···O1^iv^	0.81 (2)	2.12 (2)	2.826 (2)	146 (3)
O1*W*—H1*WB*···N3^iv^	0.81 (2)	2.53 (2)	3.218 (2)	143 (3)
O2*W*—H2*WA*···Cl2^iii^	0.83 (2)	2.36 (2)	3.190 (2)	177 (3)
O2*W*—H2*WB*···Cl2^v^	0.81 (2)	2.42 (2)	3.214 (2)	169 (3)
O3*W*—H3*WA*···Cl2	0.93 (2)	2.28 (2)	3.206 (3)	174 (3)
O3*W*—H3*WB*···O1*W*^vi^	0.90 (2)	2.25 (2)	3.146 (4)	177 (3)

Symmetry codes: (i) *x*+1, *y*, *z*; (ii) −*x*+2, −*y*+1, −*z*+1; (iii) −*x*+1, −*y*+1, −*z*; (iv) −*x*+1, −*y*+1, −*z*+1; (v) *x*, *y*+1, *z*; (vi) *x*+1, *y*−1, *z*.
