# Supplementary figures and images for: Crystal structure of (E)-3-{[2-(2,4-di­chloro­benzyl­idene)hydrazin-1-yl]carbon­yl}pyridinium chloride trihydrate
Source: Acta Crystallogr E Crystallogr Commun. 2015 Jan 10;71(Pt 2):o105–6. doi: 10.1107/S2056989015000286 (PMC4384619; doi:10.1107/S2056989015000286)

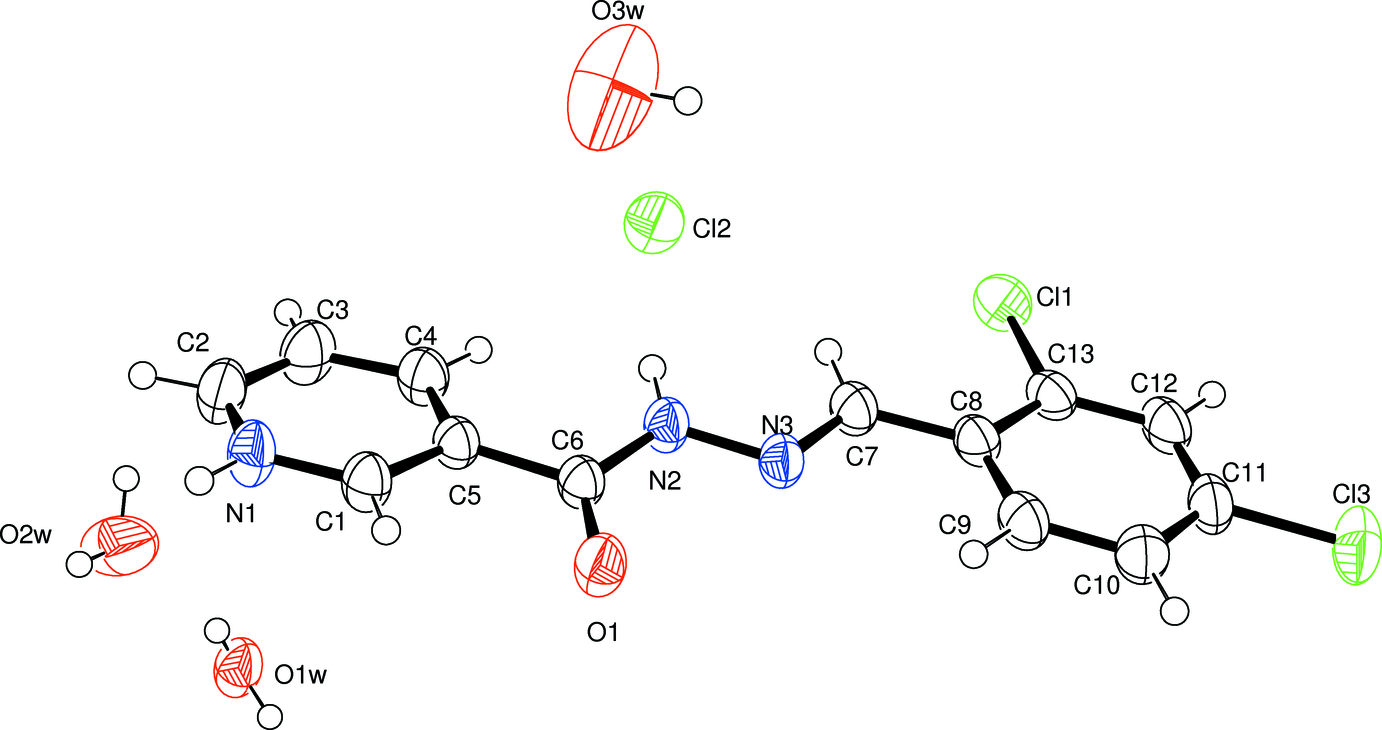

Supplement: Supplementary file 4 [file e-71-0o105-fig1.tif]

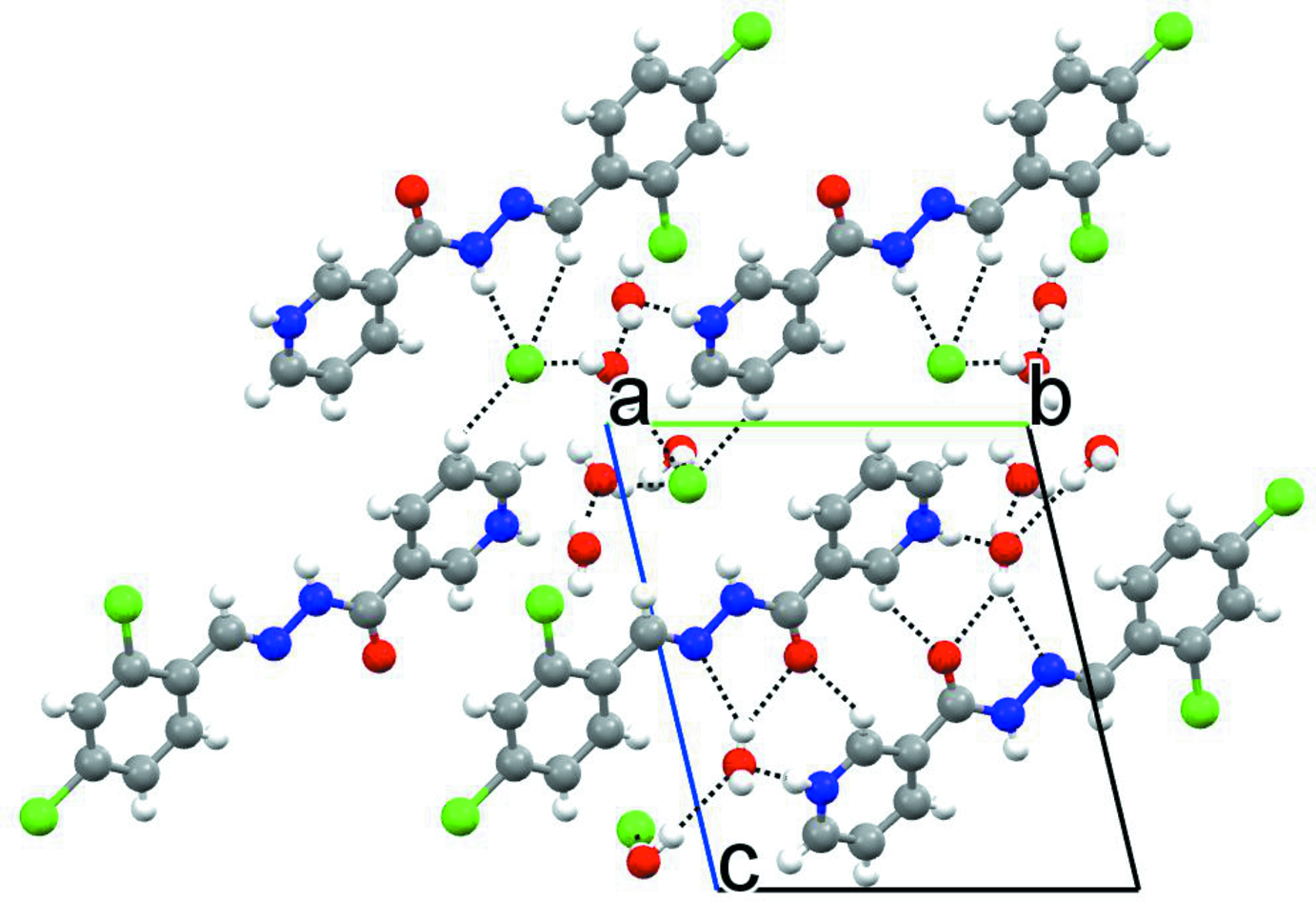

Supplement: Supplementary file 5 [file e-71-0o105-fig2.tif]

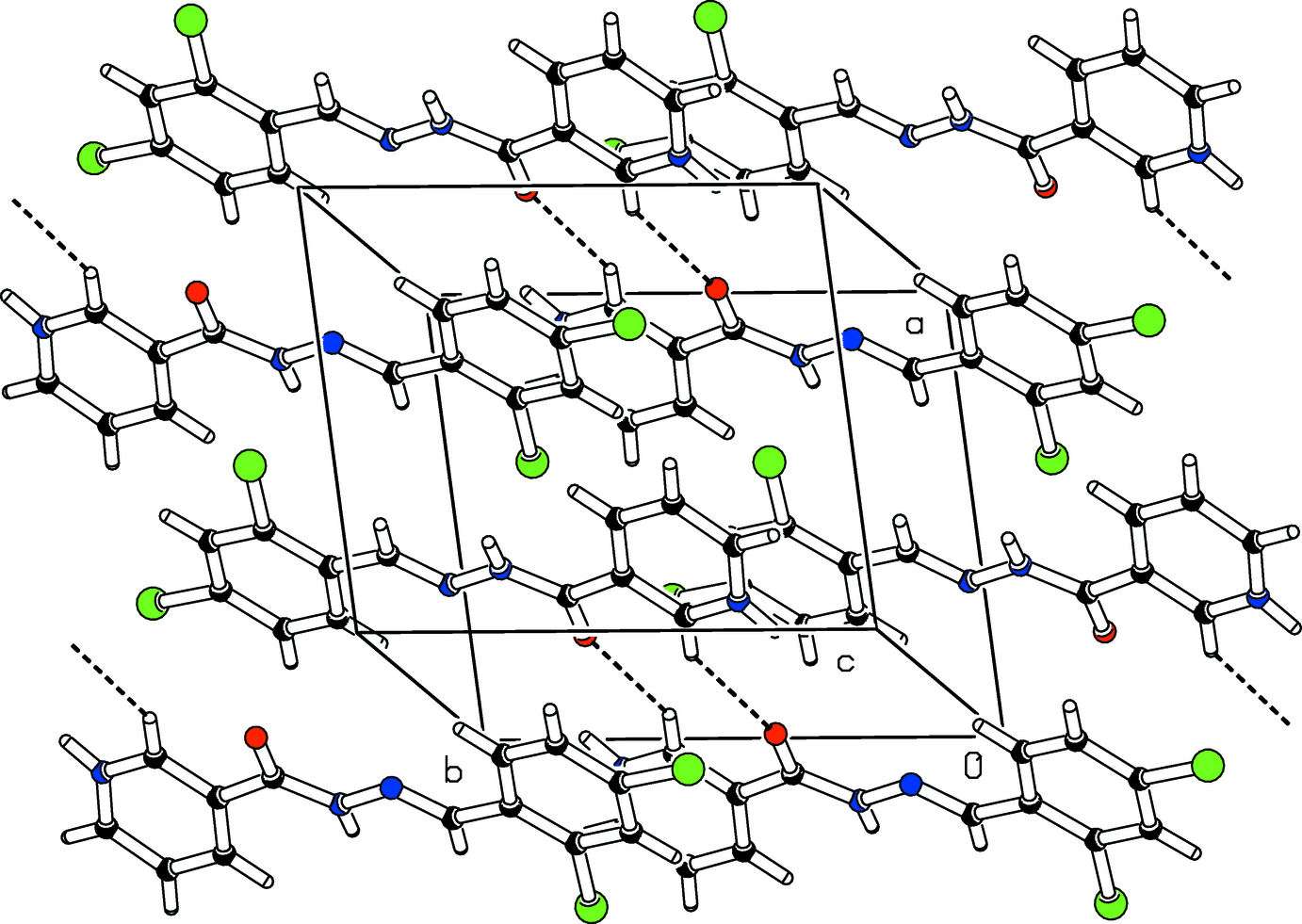

Supplement: Supplementary file 6 [file e-71-0o105-fig3.tif]
